# Gamma oryzanol impairs alcohol-induced anxiety-like behavior in mice via upregulation of central monoamines associated with *Bdnf* and *Il-1β* signaling

**DOI:** 10.1038/s41598-020-67689-w

**Published:** 2020-06-30

**Authors:** Salina Akter, Kazi Rasel Uddin, Hiroyuki Sasaki, Yijin Lyu, Shigenobu Shibata

**Affiliations:** Laboratory of Physiology and Pharmacology, School of Advanced Science and Engineering, Waseda University, Shinjuku-Ku, Tokyo, 162-8480 Japan

**Keywords:** Molecular biology, Neuroscience

## Abstract

Adolescent alcohol exposure may increase anxiety-like behaviors by altering central monoaminergic functions and other important neuronal pathways. The present study was designed to investigate the anxiolytic effect of 0.5% γ-oryzanol (GORZ) and its neurochemical and molecular mechanisms under chronic 10% ethanol consumption. Five-week-old ICR male mice received either control (14% casein, AIN 93 M) or GORZ (14% casein, AIN 93 M + 0.5% GORZ) diets in this study. We showed that GORZ could potentially attenuate alcohol-induced anxiety-like behaviors by significantly improving the main behavioral parameters measured by the elevated plus maze test. Moreover, GORZ treatment significantly restored the alcohol-induced downregulation of 5-hydroxytryptophan and 5-hydroxyindole acetic acid in the hippocampus and improved homovanillic acid levels in the cerebral cortex. Furthermore, a recovery increase in the level of 3-methoxy-4-hydroxyphenylglycol both in the hippocampus and cerebral cortex supported the anxiolytic effect of GORZ. The significant elevation and reduction in the hippocampus of relative mRNA levels of brain-derived neurotrophic factor and interleukin 1β, respectively, also showed the neuroprotective role of GORZ in ethanol-induced anxiety. Altogether, these results suggest that 0.5% GORZ is a promising neuroprotective drug candidate with potential anxiolytic, neurogenic, and anti-neuroinflammatory properties for treating adolescent alcohol exposure.

## Introduction

The high prevalence of alcohol exposure among adolescents is an important issue for public health^1–3^ worldwide. Chronic ethanol consumption during adolescence may alter the risk/reward assessment and damage the developing brain, leading to an increase in impulsivity^4^. Furthermore, underage drinking may also increase major depression risk and anxiety disorders^5,6^ later in life. In addition, central nervous monoaminergic functions are altered after adolescent ethanol exposure^7–10^ and may lead to neurological changes that reduce a person's normal fear of the consequences of their actions, and contribute to risk-taking or violent behaviors. The brain is more vulnerable to alcohol during growth and development, and many studies consistently show that teens with drinking alcohol are more likely to experience alcohol-related health problems, such as obesity, diabetes, liver and heart diseases, dementia and some forms of cancer, later in life than teens who abstain.

The most commonly used medicines for patients with mood and anxiety disorders exert their effects by modifying monoaminergic neurotransmission. In particular, selective serotonin reuptake inhibitors (SSRIs) target the serotonergic system and block the reuptake of serotonin from the synaptic cleft by inhibiting the serotonin transporter and enhancing the amount of free serotonin available. Notably, there is molecular, physiological, and clinical evidence suggesting that the serotonergic system plays pivotal role in the neural circuitry of mood and anxiety disorders^11–13^.

The functional and structural changes of the brain related with alcohol consumption and withdrawal are accompanied by changes of several cellular and molecular mechanisms. Brain-derived neurotrophic factor (BDNF) is identified as a important molecule that regulates functional changes in brain responses to alcohol use. Several reports have consistently shown that BDNF is implicated in neurogenesis^14,15^, depression^16,17^, and the synaptic changes associated with drug addiction in the adult brain^18^. In addition, oxidative stress and activation of neuroinflammatory pathways are induced by alcohol abuse^19–21^. Chronic alcohol consumption is also related with an increase in production of pro-inflammatory cytokines, such as IL1-β, due to the sensitization of immune cells^22^.

Although alcohol use disorders (metabolic and psychiatric) are leading causes of preventable death, treatment options, especially for alcohol-dependent neuropsychiatric disorders, are still limited. Therefore, there is need for encouraging natural, safe, and long-term psychiatric and physiological treatments for chronic alcohol dependence. Within this context, γ-oryzanol (GORZ), a bioactive component of brown rice that contains a mixture of ferulic acid esters and phytosterols^23^, has emerged as a therapeutic candidate. Moreover, GORZ can reach the brain as a complete structure^24,25^ and also exert several effects, including antioxidant, antiulcerogenic, antineoplastic, antidiabetic, and antiallergic properties^26–28^.

In the present study, we explored the neuropsychopharmacological activity of 0.5% GORZ by examining its effects on anxiety-like behaviors, using the elevated plus maze (EPM) test, in mice that were chronically fed alcohol during adolescence. Additionally, and more importantly, we focused on elucidating the intricate neurochemical and molecular mechanisms of GORZ, i.e., how the anxiolytic effects of GORZ were associated with central monoaminergic and neuroprotective pathways in the brain in a mouse model of chronic alcohol consumption.

## Results

All data and statistical analyses can be found in the Supplementary Table S1 online, and the schematic representation of the experiment is shown in supplementary Fig. 1.

**Figure 1:**
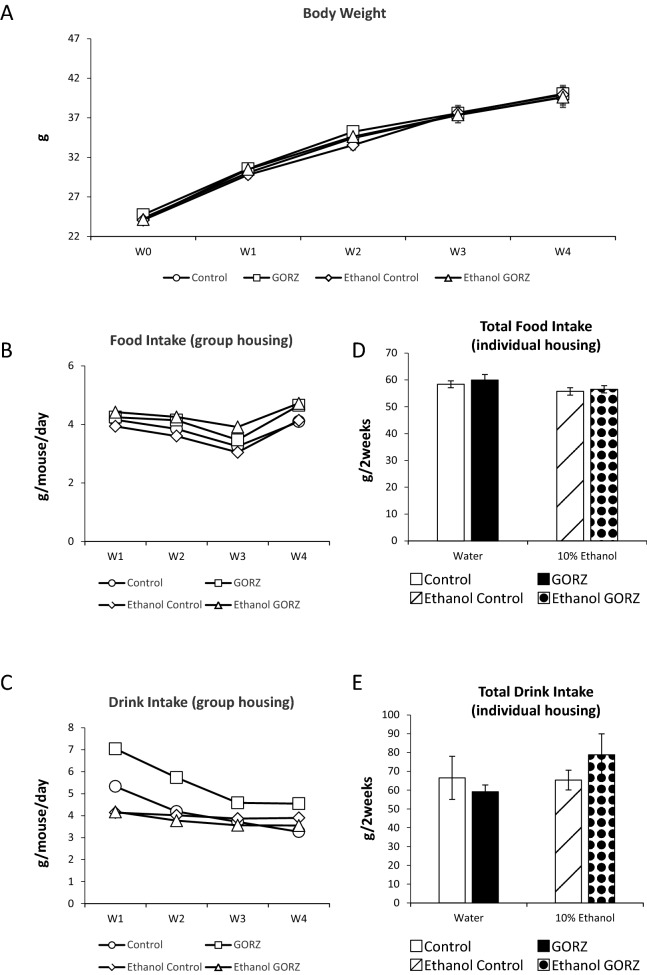
Effect of chronic 10% ethanol challenge and GORZ treatment on body weight, feed consumption and drink intake (Experiment 1). (**A**) Body weight (g) of all groups from baseline, i.e., week 0 (W0) to week 4 (W4). Data are presented as mean ± standard error (SE); n = 10–12 mice per group. (**B**) Daily food intake (g/mouse) of mice from baseline, i.e., week 0 (W0) to week 4 (W4). (**C**) Daily drink intake (g/mouse) of mice from baseline, i.e., week 0 (W0) to week 4 (W4). Data are from n = 1, because of group housing. (**D**) Total food intake and (**E**) total drink intake from week 2 to week 4 (g) by individual housing. Data are presented as mean ± standard error (SE); n = 4 mice per group.

### Chronic alcohol consumption and GORZ treatment do not affect body weight (Experiment 1)

To analyze the effect of 0.5% GORZ and 10% ethanol on body weight, mice were fed ad libitum in a group. There was no notable difference in body weight, food and drink consumption among the groups during the entire experiment period (Fig. 1A–C). As a preliminary experiment, food and drink consumption were measured under individual housing, and there were no significant differences in food and drink volumes (Fig. 1D,E). Thus, there was similar GORZ intake between the water and alcohol exposure group (Fig. 1D). In addition, there was similar alcohol intake between control non-GORZ and GORZ group (Fig. 1E).

### GORZ improves alcohol-induced anxiety-like behaviors (Experiment 2)

**Figure 2:**
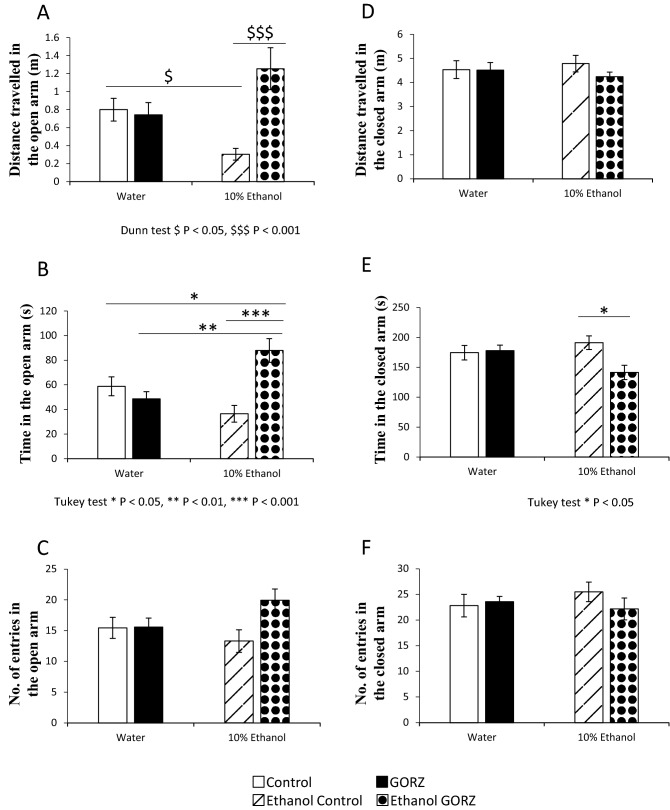
Effect of 0.5% γ-oryzanol (GORZ) treatment on chronic 10% ethanol-induced behavioral changes in the elevated plus maze (EPM) test (Experiment 2). The EPM test was conducted on W4 from ZT 5 to 7 with an ethanol wash out to measure anxiety-related behaviors. The (**A**) Distance travelled in the open arm (m), (**B**) Time in the open arm (s), (**C**) Number of entries into the open arm, (**D**) Distance travelled in the closed arm (m), (**E**) Time in the closed arm (s), and (**F**) Number of entries into the closed arm were measured. All values are expressed as mean ± standard error (SE); n = 10–12 mice per group. Two-way analysis of variance (ANOVA) with Tukey test *P < 0.05, **P < 0.01, ***P < 0.001, and Kruskal–Wallis tests followed by Dunn’s multiple comparisons test ^$^*P* < 0.05, ^$$$^*P* < 0.001 were used to determine significance.

In the EPM test, anxiety-like behaviors induced by chronic 10% ethanol drinking were confirmed, because the distance travelled in the open arms (m) was significantly (*P* < 0.05) lower in the ethanol-exposed control group than in the water-exposed control group (Fig. 2A). However, 0.5% GORZ treatment significantly increased the distance travelled in the open arms (m) (*P* < 0.001) and, remarkably, attenuated the anxiety symptoms caused by chronic ethanol consumption (Fig. 2A). A significant increase in the amount of time spent in the open arms (s) of the EPM was observed in the ethanol-exposed GORZ-treated mice, as compared to the ethanol-exposed control group (*P* < 0.001), the water-exposed GORZ group (*P* < 0.01), and the water-exposed control group (*P* < 0.05) (Fig. 2B). Additionally, the number of entries in the open arm was slightly improved by GORZ treatment although a significant difference was not observed even after chronic ethanol consumption (Fig. 2C).

Simultaneously, behavioral parameters in the closed arm of EPM displayed the opposite pattern (Fig. 2D–F). A significant decrease in the time spent in the closed arms (s) was observed in the ethanol-exposed GORZ-treated mice compared with the ethanol-exposed control group (*P* < 0.05) (Fig. 2E). When the total traveling distance was calculated in each group, there were no significance differences among the groups (5.33 ± 0.39 m/5 min for water exposed control; 5.25 ± 0.39 for water exposed GORZ; 5.09 ± 0.34 for ethanol-exposed control; and 5.49 ± 0.23 for ethanol-exposed GORZ), suggesting that the anxiolytic effect of GORZ in the open arms was not due to the decrease of locomotor activity.

Thus, the EPM test results supported the hypothesis that chronic ethanol exposure during adolescence induces anxiety-like behaviors, which could be profoundly reversed by the anxiolytic effect of 0.5% GORZ.

### GORZ restores the chronic alcohol-induced downregulation of central monoamine neurotransmitters and their metabolites in the hippocampus and cerebral cortex (Experiment 3)

**Figure 3:**
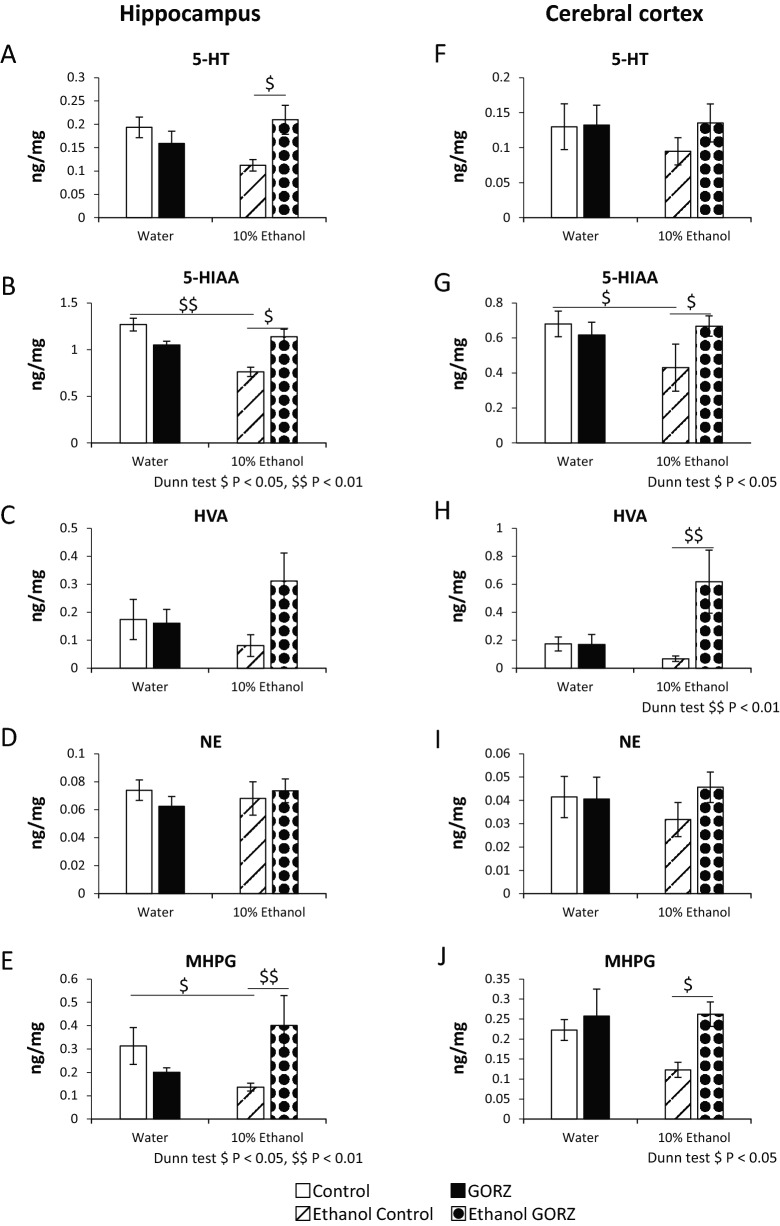
Effect of 0.5% γ-oryzanol (GORZ) on the levels of monoamine neurotransmitters and their metabolites in the hippocampus (**A–E**), and cerebral cortex (**F–J**) of mice exposed to chronic ethanol for 5 weeks during adolescence (Experiment 3). All values are expressed as mean ± standard error (SE); n = 10–14 mice per group. Statistical differences were evaluated using non-parametric analyses with Kruskal–Wallis tests followed by Dunn's multiple comparisons test ^$^*P* < 0.05, ^$$^*P* < 0.01, ^$$$^*P* < 0.001.

Figure 3A–J shows the differences in the levels of monoamine neurotransmitters and their metabolites in the hippocampus and cerebral cortex of the brain between the groups. The results indicate that ethanol-exposed control mice had a significant decrease in 5-hydroxyindole acetic acid (5-HIAA) levels in the hippocampus (*P* < 0.01) and cerebral cortex (*P* < 0.05), compared to the water-exposed control mice; therefore, the anxiety level would likely be higher in ethanol-exposed control mice (Fig. 3B,G). Interestingly, GORZ treatment reversed the ethanol-induced decreases in 5-HIAA in the hippocampus and cerebral cortex and attenuated anxiety (Fig. 3B,G). The level of 5-hydroxytrypatamine (5-HT) was also significantly improved in GORZ-treated mice (*P* < 0.05) versus untreated mice that chronically consumed ethanol (Fig. 3A). No significant differences were found in the hippocampal level of norepinephrine (NE) among the groups (Fig. 3D).

Compared to water-exposed control mice, ethanol-exposed control mice had a slightly lower level of the main metabolite of dopamine (DA), homovanillic acid (HVA), in the cerebral cortex, which was significantly reversed (*P* < 0.01) after GORZ treatment (Fig. 3H). GORZ treatment is also expected to exert an anxiolytic effect in mice due to the increase of 3-methoxy-4-hydroxyphenylglycol (MHPG) level both in the hippocampus (*P* < 0.01) and cerebral cortex (*P* < 0.05) during chronic ethanol exposure (Fig. 3E,J). However, there was no effect of ethanol exposure and GORZ treatment on 5-HT and NE levels in the cerebral cortex of mice (Fig. 3F,I).

Therefore, 0.5% GORZ may reduce alcohol-induced anxiety-like behaviors via upregulation of the central monoaminergic system in the hippocampus and cerebral cortex.

### GORZ treatment regulates *Bdnf* and *Il-1β* signaling in the hippocampus to reduce chronic alcohol-induced anxiety (Experiment 4)

**Figure 4:**
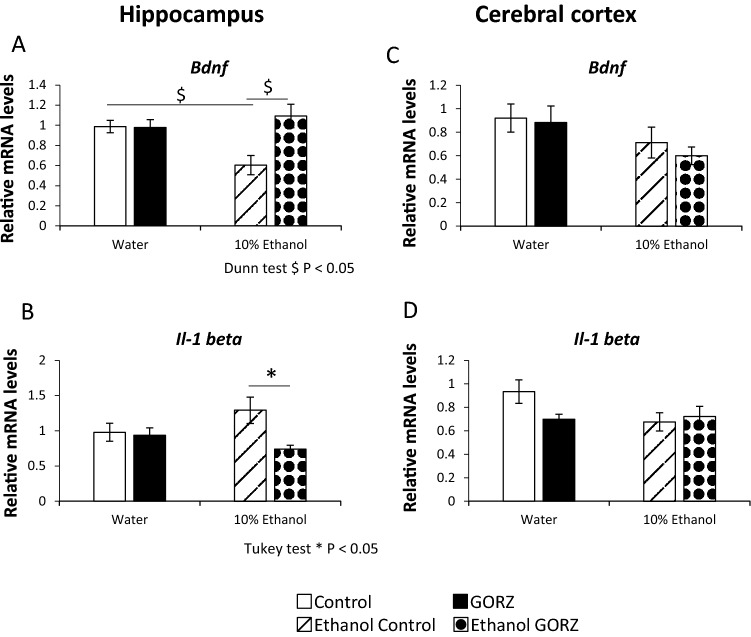
Effect of 0.5% γ-oryzanol (GORZ) treatment on *Bdnf* and *Il-1β* mRNA expression in the hippocampus (**A**, **B**) and cerebral cortex (**C**, **D**) of mice exposed to chronic ethanol for 5 weeks during adolescence (Experiment 4). All values are expressed as mean ± standard error (SE); n = 9–11 mice per group. Statistical differences were evaluated using two-way analysis of variance (ANOVA) with Tukey test *P < 0.05, and Kruskal–Wallis tests followed by Dunn’s multiple comparisons test. ^$^*P* < 0.05.

To gain insight into a possible neural mechanism of 0.5% GORZ that could mediate chronic alcohol consumption-induced anxiety-like behaviors, we examined *Bdnf* mRNA expression in the hippocampus and cerebral cortex (Fig. 4A,C). Our results showed a significant suppression (*P* < 0.05) of hippocampal *Bdnf* mRNA levels in the ethanol-exposed control group when compared to water-exposed control mice. More importantly, GORZ-treated mice showed a marked increase (*P* < 0.05) in hippocampal *Bdnf* mRNA levels, even during chronic ethanol-induced anxiety (Fig. 4A).

The relative mRNA level of the inflammatory cytokine, *Il-1β*, was also examined in the hippocampus and cerebral cortex (Fig. 4B,D) in order to elucidate the intricate anti-inflammatory mechanism by which 0.5% GORZ regulates alcohol-induced anxiety. We did not observe any significant influence of chronic 10% ethanol exposure on the relative mRNA levels of *Il-1β* in either the hippocampus or cerebral cortex (Fig. 4B,D). However, we did note that 0.5% GORZ treatment significantly reduced (*P* < 0.05) *Il-1β* mRNA levels in the hippocampus (Fig. 4B) in the chronic ethanol consumption group. However, GORZ showed a minor response regarding *Bdnf* and *Il-1β* mRNA levels in cerebral cortex (Fig. 4C,D).

This finding suggests that the regulation of *Bdnf* and *Il-1β* signaling by 0.5% GORZ treatment promotes possible neural mechanisms in the hippocampus that mediate chronic alcohol-induced anxiety-like behaviors.

### GORZ shows a minor effect on systemic BDNF and IL-1β levels during chronic alcohol-induced anxiety (Experiment 4)

**Figure 5:**
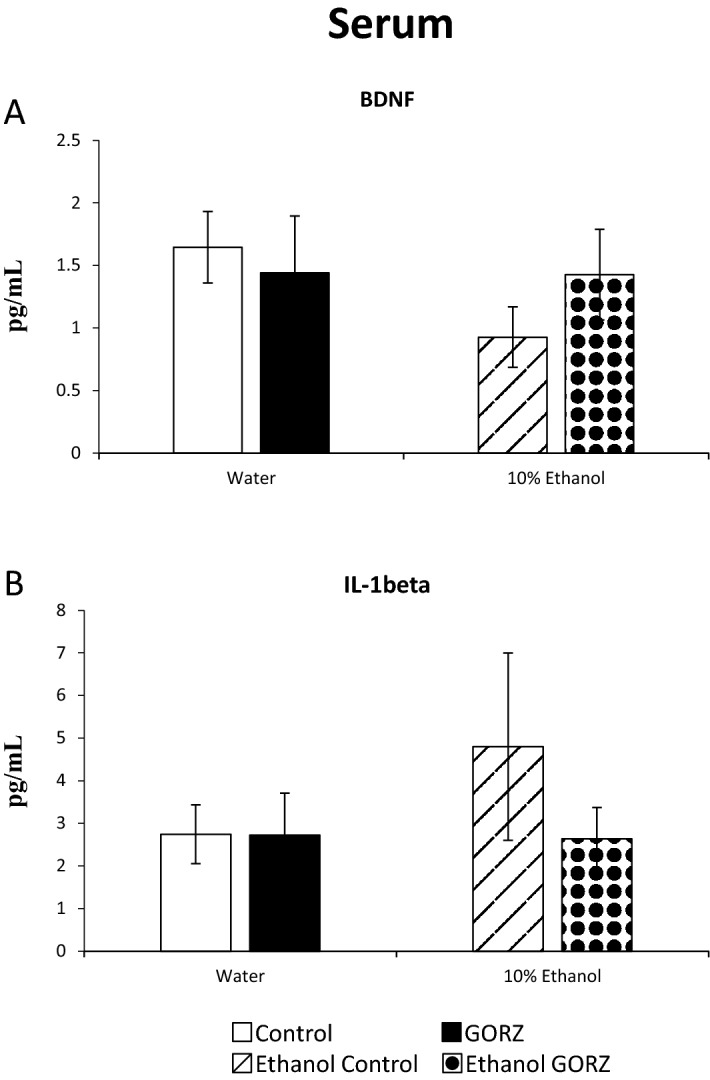
Effect of 0.5% γ-oryzanol (GORZ) treatment on the systemic BDNF and IL-1β concentrations (**A**, **B**) of mice exposed to chronic ethanol for 5 weeks during adolescence (Experiment 4). All values are expressed as mean ± standard error (SE); n = 5–7 mice per group.

Two important biomarkers, BDNF and IL-1β in serum were assayed to establish a link of the regulatory mechanism of 0.5% GORZ treatment between the central nervous system (CNS) and the circulatory system under alcohol-induced anxiety (Fig. 5A,B). Here, we showed that, both chronic 10% ethanol exposure and GORZ treatment affect the systemic BDNF concentrations without any significance (Fig. 5A). The ethanol-exposed control group also displayed a slight increase in serum IL-1β concentration compared to water-exposed control mice. GORZ treatment attenuated the increase in the pro-inflammatory marker in the circulatory system, though this effect did not reach statistical significance (Fig. 5B).

Thus, the neuroprotective effects of 0.5% GORZ may require more than 5 weeks of treatment to result in meaningful changes in the circulatory system and reduce alcohol-induced anxiety.

## Discussion

Studies addressing the impact of alcohol-induced anxiety due to monoaminergic malfunction are of crucial concern. To the best of our knowledge, this study is the first to investigate the effects of GORZ treatment on the endogenous monoaminergic system and associated neuronal pathways in an animal model of alcoholic stress induced anxiety. In the present study, mice were exposed to chronic 10% ethanol during adolescence, and a comparative assessment using similar feed consumption and drink intake was conducted in the experimental period to evaluate changes in body weight during adolescence. We found no relationship between the effect of alcohol calories and body weight, and our data were in agreement with a previous report^29^.

In this experimental design, mice were housed in each cage as a treatment group, each constituting an experimental unit. Therefore, we were not able to measure feed consumption and drink intake per mice. However, GORZ did not affect food and ethanol consumption by individually measurement, as a preliminary experiment (Fig. 1D,E).

Natural stimuli (e.g., fear of novel open spaces and fear of balancing on a relatively narrow, raised platform) in the EPM makes the EPM test the most valid ethological animal model of anxiety^30^. Studies have also shown that alcohol abuse causes anxiety, depression and cognitive impairment in animal models^31,32^. Behavior evaluation in the present study indicates that chronic ethanol exposure induces anxiety-like effects by significantly reducing the distance travelled in the open arms (m) of the EPM. Interestingly, GORZ treatment can significantly ameliorate this anxiety-like behavior in adolescent mice by increasing the distance traveled (m) and time (s) spent in the open arms of the EPM. In addition, GORZ treatment significantly decreased the time (s) spent in the closed arm of the EPM. Thus, the EPM test results indicate an anxiolytic effect of 0.5% GORZ in adolescent ethanol exposure.

In the EPM test, the illumination level was maintained at 100–150 lx as some previous reports have addressed the exploratory behavior of rodents in the elevated plus-maze according to the ranges of light intensities^33–35^. Moreover, it is reported that low-intensity illumination causes reduction of avoidance in the open arm. Thus, low-intensity illumination (5–30 lx) should be preferred to analyze an anxiogenic effect, whereas an anxiolytic effect should be induced under high-intensity illumination (200–400 lx or more)^36–39^. In the current experiment, we evaluated the anxiety behavior by EPM test, however, in the future experiments, we should use multiple anxiety behavior tests such as open field test and fear conditioning test. After the EPM test which was followed with a short duration (2 h) of a washout with water from ethanol, mice were again kept under the same experimental condition for a week to continue the chronic 10% ethanolic stress situations and then sacrificed. Therefore, the changes in central monoamines, BDNF, and IL-1β signaling in ethanol-exposed mice were caused by chronic ethanol exposure rather than ethanol withdrawal.

The central monoaminergic system, including 5-HT and NE has been widely involved in the pathophysiology and therapeutic measures for behavioral effects and emotional disorders^40–42^. It is widely reported that impairment of the monoaminergic system may be a promising mechanism that drives the anxiety-related pathophysiology^43–45^. In addition, the synthesized and released neurotransmitters in the cerebral cortex and hippocampus are believed to be involved in anxiety disorders^46,47^. Alcohol has been identified by many reports as a big risk factor for neuropsychiatric disorders and is also related with monoamine malfunction^48,49^. The present study is consistent with these reports. Our study shows that anxiety following chronic ethanol consumption is linked with an impaired central monoaminergic system in the hippocampus, as indicated by significant decreases in 5-HIAA and in MHPG levels, and in cerebral cortex, by a significant decrease in 5-HIAA and a slight decrease in MHPG. Furthermore, the treatment with SSRIs and serotonin and NE reuptake inhibitors improve anxiety symptoms, and this improvement is one explanation that supports the dysfunction of serotonergic, noradrenergic, and, occasionally, dopaminergic neurons in anxiety patients^50^. Moreover, the hippocampus is brain area involved in mood and anxiety disorders^51^. Based on this evidence, we elucidated how the anxiolytic-like effects of GORZ were linked to monoamines. Interestingly, 0.5% GORZ could reverse the alcohol-induced anxiety-like effects during adolescence by significantly elevating 5-HIAA and MHPG in the hippocampus, and HVA and MHPG in the cerebral cortex. Furthermore, an increase in the level of MHPG, an important NE metabolite both in the hippocampus and cerebral cortex supported the anxiolytic effect of 0.5% GORZ in chronic ethanol drinking condition. We evaluated the content of neurotransmitters and also their metabolites only in the hippocampus and cerebral cortex, because many studies involving anxiety focused on the hippocampus and cerebral cortex in the brain^46,47^. However, the amygdala also plays a pivotal role in changing the levels of monoamines, such as NE and serotonin, in anxiety, using several animal models^35,52^. The neurotransmitter synthesis and release in the amygdala should also be examined in future studies to estimate whether the neurotransmitter changes in the amygdala are similar to the results of our current study.

Alcohol is a neurodegenerative agent with sedative properties^53,54^. In addition, alcohol affects neurogenesis by decreasing BDNF activities that promote cell survival^55,56^. An important nerve growth factor, BDNF, has been reported to play an important role in synaptic plasticity, neurogenesis, and neuronal survival^57^, and is associated with the behavioral effects caused by addictive drugs^58^. BDNF also plays a crucial role in the maintenance of important brain regions that are related to the regulation of behavior and cognition. BDNF increases brain plasticity through the action of the ligand-like specific binding to tropomyosin receptor kinase B (TrkB) and the mediation of different neurotropic signaling cascades. Our current results indicate that, chronic ethanol consumption leads to a nonsignificant trend toward impaired *Bdnf* relative mRNA expression in the hippocampus and cerebral cortex. Several reports have examined neurotrophin expression after adolescent alcohol consumption, which also differed^59–62^ owing to the differences in each alcohol paradigm, examined age, and/or the stage of the disease process. However, GORZ treatment in the diet has a significant augmentation of the *Bdnf* mRNA expression in the hippocampus. Hence, a crucial finding in the present study is that 0.5% GORZ effectively mediates alcohol-induced anxiety-like symptoms by improving *Bdnf* signaling in the hippocampus.

Patients with mood and anxiety disorders may have higher levels of the inflammatory cytokine, IL-1β. Several previous papers reported the elevation of systemic IL-1β levels^63,64^, while other paper did not found such association of IL-1β^65^, or other neuroimmune biomarkers. In the present study, we found no relationship between alcohol-induced anxiety and levels of measured *Il-1β* mRNA in either the hippocampus or cerebral cortex. If different types of trauma yield different immunological responses, such mixed findings may be observed. However, in this study, GORZ treatment significantly reduced *Il-1β* mRNA expression in the hippocampus of mice that consumed alcohol chronically. Therefore, in the current study, 0.5% GORZ is shown to be a natural, bioactive component with potential neuroprotective and anti-neuroinflammatory properties that preferentially targets the hippocampus, rather than the cerebral cortex.

The mechanism by which GORZ regulates BDNF and IL-1β signaling is not fully understood. Recently, the release of BDNF was related with cellular depolarization and intra- and extracellular changes of Ca^2+^ ion concentrations through the activation of IP_3_^66^. BDNF is also modulated by adenylate cyclase-coupled receptors. Another possible mechanism modulated by GORZ is through the signaling pathways of CREB and ERK1/2 molecules, and activation of these pathways causes neurotropic factors production^67^. The main source of circulating BDNF is from the brain, and activation of the brain results in elevation of BDNF concentrations that can be detectable in the internal jugular vein^68,69^. Consistently, decrease levels of serum BDNF are related with aggravation and poor outcomes in diseases with neurodegeneration^70^. A possible mechanism that underlies the ability of GORZ to ameliorate the alcohol-induced changes in the hippocampal mRNA expression of *Il-1β* could be the signaling cascade of PPAR-α: n–3 LCPUFA-dependent PPAR-α activation stimulates its interaction with NF-κB p65 subunit to produce the PPAR-α/NF-κB complex, leading to decrease in mRNA expression in the proinflammatory genes stimulated by NF-κB^71^. Here, we found a similar trend between alcohol-induced anxiety with systemic and hippocampal BDNF and IL-1β levels. In addition, GORZ played a positive role in the circulatory system, although it was not statistically significant. As IL-1β is synthesized as a pro-protein that must be cleaved before its release into the circulatory system, mRNA expression of *Il-1β* in the hippocampus and cerebral cortex is not necessarily associated with systemic inflammation^72^.

In summary, the present study determined that the anxiolytic effects of 0.5% GORZ could be associated with improvements of monoamines, *Bdnf* signaling, and downregulation of IL-1β signaling in the brain. This study has furthered the elucidation of the intricate pathophysiological mechanisms of anxiety in a mouse model with chronic alcohol consumption and has evaluated a potential therapeutic drug.

## Materials and methods

### Animals

Five-week-old, male ICR mice (Tokyo Laboratory Animals Science Co., Ltd., Tokyo, Japan) were used in these experiments. Supplementary Fig. 1 (online) shows a schematic representation of the experimental design and protocol. Mice were weight matched and randomly divided into four groups (Control, GORZ, Ethanol Control and Ethanol GORZ). Mice were kept under a reverse 12:12 light/dark cycle (with lights on at 20:00 h) with constant temperature and humidity (22 ± 2 °C, 60 ± 5%) under light intensity of 100–150 lx. Zeitgeber time (ZT) 0 was lights-on time, and ZT12 was light-off time, respectively. Food and drink were available ad libitum. The procedures conformed to the “Fundamental Guidelines for Proper Conduct of Animal Experiment and Related Activities in Academic Research Institutions” (published by the Ministry of Education, Culture, Sports, Science and Technology, Japan), and were approved by the Committee for Animal Experimentation of the School of Science and Engineering at Waseda University (permission # 2017-A074, 2018-A010).

### Dietary treatment and ethanol challenge

We supplied mice control food (14% casein, AIN 93 M formula; Oriental Yeast Co. Ltd., Tokyo, Japan) and GORZ food (the mouse standard diet 14% AIN-93 M combined with 0.5% GORZ (Sigma-Aldrich, St. Louis, MO, USA). The reason for the dose of GORZ chosen for current experiment has been described in our previous paper^35^. Mice were also given ad libitum access to tap water or 10% ethanol during the experiment period.

### Feeding schedule

In the experiments, mice were maintained using ad libitum feeding for 24-h for a total 5 weeks. The food and drinking bottled water were changed once a week; however, when the bottled water for drinking included 10% ethanol, it was changed twice a week to avoid any fermentation during the experimental period. We prepared two bottles so as not to lose the opportunity to drink ethanol because of group housing. The mouse housing cage was cleaned with fresh wood shavings every week. The body weight, feed consumption, and drink intake of the mice were monitored weekly. A total of 10–12 mice were housed as an experimental treatment group. This is the reason why we measured feed consumption and drink intake as a group. However, in order to check food and drink consumption in each mouse, we measured individually the food and drink volumes, as preliminary experiments (Fig. 1D,E).

### Elevated plus maze test

After 4 weeks of ad libitum feeding, ethanol-induced anxiety-like behaviors were assessed using the EPM test for a 5-min period for each mouse, which is the most common test of anxiety in rodents^73,74^, at ZT 4 to 5 following a 2-h washout with water from ethanol. This recovery time was allowed to avoid the interference between chronic ethanolic stress and behavioral test. In addition, feeding and drinking patterns showed the peaks occurring around at early half of dark period. So, we expected that there may be no direct ethanol effect on behavioral experiment at ZT4-ZT5 (middle of light period).

The details of the EPM apparatus and test protocols have been published in our previous paper^35^. Behavioral parameters, such as staying time (s) in the open and closed arms, moving distance (m) in the open and closed arms, and entering number (n) in the open and closed arms, were measured and analyzed using software named ANY-MAZE (Stoelting, IL, USA). ANY-MAZE judges the mouse entry into each arm, when the center body but not front or hind legs of the mouse enters the open or closed arm. The light intensity above EPM apparatus was fixed at 100–150 lx. The apparatus was cleaned with 70% ethanol after each trial of the EPM test to avoid mouse smell.

### Animal euthanasia and sample collection

At the end of 5 weeks' chronic dietary treatment following a week same experimental condition after the EPM test, mice were anesthetized using isoflurane and sacrificed at ZT 5.0 and blood samples were collected from the neck, and collected samples were kept at room temperature for 1 h; to obtain serum, the samples were centrifuged at 3,000 rpm for 15 min, and serum was stored at − 80 °C until further analysis. In contrast, whole brains were rapidly removed, and 2-mm thick brain slices including the hippocampus and cerebral cortex were frontally sliced using a brain matrix (# 0530; Bioresearch Center Co., Nagoya, Japan). The hippocampal and cerebral cortices including slices were dissected by a thin blade, based on the neuroanatomical landmarks from the brain atlas, corresponding approximately to bregma − 0.5 to bregma − 2.5^75^. A free-hand dissection by a small scissor was performed to separate the dorsal part of hippocampus (mean wet weight, 14.3 mg) from the parietal region of cerebral cortex (mean wet weight, 17.2 mg). The tissues of the hippocampal and cerebral cortices were immediately stored at − 80 °C until further analysis. Our previous study demonstrated the detailed protocol^76^.

### High-performance liquid chromatography-electrochemical detection (HPLC-ECD)

To explore detailed neurochemical mechanisms involved in ethanol associated anxiety and a possible treatment strategy, the levels of central monoamine neurotransmitters and their metabolites, such as 5-HT/5-HIAA, DA/HVA, and NE/MHPG, were detected in the hippocampus and cerebral cortex using HPLC-ECD (HTEC 500; Eicom, Kyoto, Japan) according to a previously published protocol^35^. DA contents were too small to detect in the hippocampus and cerebral cortex.

### Real-time reverse transcription polymerase chain reaction (RT-PCR)

The levels of relative mRNA levels of *Bdnf* and *Il1-β* were detected in the hippocampus and cerebral cortex by quantitative (q)-reverse transcription polymerase chain reaction (RT-PCR) using One-Step SYBR RT-PCR Kit (Takara Bio Inc., Shiga, Japan) with specific primer pairs (for list of primers, see Supplementary Table S2 online) on a Piko Real PCR system (Thermo Fisher Scientific, Waltham, MA, USA). Total RNA was extracted using TRIzol Reagent (Ambion, Oakland, USA) following with the manufacturer’s protocol. Total RNA content was evaluated by the NanoVue Plus spectrophotometer (GE Healthcare Life Sciences, Piscataway, NJ, USA). The primers were designed using by Primer 3 software^77,78^. qRT-PCR was carried out under the following conditions. cDNA was synthesized at 42 °C for 15 min and followed by 95 °C for two min. PCR was amplificated for 40 cycles with denaturation at 95 °C for five s, and followed with annealing and extension at 60 °C for 20 s. The relative expression of target genes was normalized to that of *Gapdh* mRNA. Data were analyzed using the ΔΔCt method. A melt curve analysis of each primer was performed to identify non-specific products. Our previous study demonstrated the detailed protocol^79^.

### Enzyme-linked immunosorbent assay

Levels of serum BDNF and IL1-β were measured through an enzyme-linked immunosorbent assay (ELISA), using a commercial ELISA kit (Biosciences, The Barton Sa, Australia; R&D Systems, Inc, MN, USA, respectively) according to the manufacturer’s instructions. For BDNF, Absorbance was measured at 450 nm for detection of BDNF levels. Absorbance was measured at 450 and 570 nm for IL1-β. The readings at 570 nm were subtracted from those at 450 nm to correct the optical noise.

### Statistical analysis

Data are expressed as the mean ± standard error of mean (SEM). Statistical analyses were performed using Graph Pad Prism version 6.03 (Graph Pad Software, San Diego, CA). At first, we determined whether the data showed a normal distribution and variation with/without equal, using the D’Agostino-Pearson test and the Bartlett’s test, respectively. Parametric analyses were conducted using a two-way analysis of variance (ANOVA) with Sidak test (if the interaction factor is not significant) and Tukey test (if the interaction factor is significant) for post-hoc analysis. If normality was absent, non-parametric analyses were conducted with Kruskal–Wallis tests, and Dunn's multiple comparisons test was conducted as post-hoc comparison. Statistical differences were considered significant when the *P* < 0.05.

### Ethics approval

The Committee for Animal Experimentation of the School of Science and Engineering at Waseda University (permission # 2017-A074) approved the current animal experimental protocol.

## Supplementary information

Supplementary information

## References

[1] Johnston, L. D., O'Malley, P. M., Miech, R. A., Bachman, J. G., Schulenberg, J. E. Monitoring the future national survey results on drug use: 1975–2013: overview, key findings on adolecent drug use. *Institute for Social Research, The Unversity of Michigan, Ann Arbor*, 88 (2014).

[2] Schuckit MA (2014). Predictors of subgroups based on maximum drinks per occasion over six years for 833 adolescents and young adults in COGA. J. Stud. Alcohol Drugs.

[3] Windle M (2008). Transitions into underage and problem drinking: Developmental processes and mechanisms between 10 and 15 years of age. Pediatrics.

[4] White HR (2011). Associations between heavy drinking and changes in impulsive behavior among adolescent boys. Alcohol. Clin. Exp. Res..

[5] Boden JM, Fergusson DM (2011). Alcohol and depression. Addiction.

[6] Briere FN, Rohde P, Seeley JR, Klein D, Lewinsohn PM (2014). Comorbidity between major depression and alcohol use disorder from adolescence to adulthood. Compr. Psychiatry.

[7] Badanich KA, Maldonado AM, Kirstein CL (2007). Chronic ethanol exposure during adolescence increases basal dopamine in the nucleus accumbens septi during adulthood. Alcohol. Clin. Exp. Res..

[8] Pascual M, Boix J, Felipo V, Guerri C (2009). Repeated alcohol administration during adolescence causes changes in the mesolimbic dopaminergic and glutamatergic systems and promotes alcohol intake in the adult rat. J. Neurochem..

[9] Philpot RM, Wecker L, Kirstein CL (2009). Repeated ethanol exposure during adolescence alters the developmental trajectory of dopaminergic output from the nucleus accumbens septi. Int. J. Dev. Neurosci..

[10] Wedekind D (2010). Serotonergic function, substance craving, and psychopathology in detoxified alcohol-addicted males undergoing tryptophan depletion. J. Psychiatr. Res..

[11] Paul ED, Lowry CA (2013). Functional topography of serotonergic systems supports the Deakin/Graeff hypothesis of anxiety and affective disorders. J. Psychopharmacol..

[12] Ressler KJ, Nemeroff CB (2000). Role of serotonergic and noradrenergic systems in the pathophysiology of depression and anxiety disorders. Depress. Anxiety.

[13] Ciarleglio CM, Resuehr HE, McMahon DG (2011). Interactions of the serotonin and circadian systems: Nature and nurture in rhythms and blues. Neuroscience.

[14] Lee E, Son H (2009). Adult hippocampal neurogenesis and related neurotrophic factors. BMB Rep..

[15] Shen K, Cowan CW (2010). Guidance molecules in synapse formation and plasticity. Cold Spring Harb. Perspect. Biol..

[16] Duman RS, Monteggia LM (2006). A neurotrophic model for stress-related mood disorders. Biol. Psychiatry.

[17] Neto FL, Borges G, Torres-Sanchez S, Mico JA, Berrocoso E (2011). Neurotrophins role in depression neurobiology: A review of basic and clinical evidence. Curr. Neuropharmacol..

[18] Pandey SC, Zhang H, Roy A, Misra K (2006). Central and medial amygdaloid brain-derived neurotrophic factor signaling plays a critical role in alcohol-drinking and anxiety-like behaviors. J. Neurosci..

[19] Crews FT (2008). Alcohol-related neurodegeneration and recovery: Mechanisms from animal models. Alcohol Res. Health.

[20] Das SK, Vasudevan DM (2007). Alcohol-induced oxidative stress. Life Sci..

[21] Haorah J (2008). Mechanism of alcohol-induced oxidative stress and neuronal injury. Free Radic. Biol. Med..

[22] Crews FT (2006). Cytokines and alcohol. Alcohol. Clin. Exp. Res..

[23] Lerma-García MJ, Herrero-Martínez JM, Simó-Alfonso EF, Mendonça CRB, Ramis-Ramos G (2009). Composition, industrial processing and applications of rice bran γ-oryzanol. Food Chem..

[24] Kozuka C (2015). gamma-Oryzanol protects pancreatic beta-cells against endoplasmic reticulum stress in male mice. Endocrinology.

[25] Kozuka C, Yabiku K, Takayama C, Matsushita M, Shimabukuro M (2013). Natural food science based novel approach toward prevention and treatment of obesity and type 2 diabetes: Recent studies on brown rice and gamma-oryzanol. Obes. Res. Clin. Pract..

[26] Islam MS (2011). Biological abilities of rice bran-derived antioxidant phytochemicals for medical therapy. Curr. Top. Med. Chem..

[27] Goufo P, Trindade H (2014). Rice antioxidants: Phenolic acids, flavonoids, anthocyanins, proanthocyanidins, tocopherols, tocotrienols, gamma-oryzanol, and phytic acid. Food Sci. Nutr..

[28] Kim SP, Kang MY, Nam SH, Friedman M (2012). Dietary rice bran component gamma-oryzanol inhibits tumor growth in tumor-bearing mice. Mol. Nutr. Food Res..

[29] Yeomans MR (2010). Alcohol, appetite and energy balance: Is alcohol intake a risk factor for obesity?. Physiol. Behav..

[30] Dawson GR, Tricklebank MD (1995). Use of the elevated plus maze in the search for novel anxiolytic agents. Trends Pharmacol. Sci..

[31] Hermens DF (2013). Pathways to alcohol-induced brain impairment in young people: A review. Cortex.

[32] Heinz AJ, Beck A, Meyer-Lindenberg A, Sterzer P, Heinz A (2011). Cognitive and neurobiological mechanisms of alcohol-related aggression. Nat. Rev. Neurosci..

[33] Komada M, Takao K, Miyakawa T (2008). Elevated plus maze for mice. J. Vis. Exp..

[34] Okonogi T, Nakayama R, Sasaki T, Ikegaya Y (2018). Characterization of peripheral activity states and cortical local field potentials of mice in an elevated plus maze test. Front. Behav. Neurosci..

[35] Akter S (2018). Anxiolytic effects of gamma-oryzanol in chronically- stressed mice are related to monoamine levels in the brain. Life Sci..

[36] Bertoglio LJ, Carobrez AP (2002). Behavioral profile of rats submitted to session 1-session 2 in the elevated plus-maze during diurnal/nocturnal phases and under different illumination conditions. Behav. Brain Res..

[37] Cosquer B, Galani R, Kuster N, Cassel JC (2005). Whole-body exposure to 2.45 GHz electromagnetic fields does not alter anxiety responses in rats: A plus-maze study including test validation. Behav. Brain Res..

[38] Pereira LO, da Cunha IC, Neto JM, Paschoalini MA, Faria MS (2005). The gradient of luminosity between open/enclosed arms, and not the absolute level of Lux, predicts the behaviour of rats in the plus maze. Behav. Brain Res..

[39] Leo LM (2014). Age-dependent relevance of endogenous 5-lipoxygenase derivatives in anxiety-like behavior in mice. PLoS ONE.

[40] Millan MJ (2004). The role of monoamines in the actions of established and "novel" antidepressant agents: A critical review. Eur. J. Pharmacol..

[41] Bondi CO, Jett JD, Morilak DA (2010). Beneficial effects of desipramine on cognitive function of chronically stressed rats are mediated by alpha1-adrenergic receptors in medial prefrontal cortex. Prog. Neuropsychopharmacol. Biol. Psychiatry.

[42] Arnsten AF, Li BM (2005). Neurobiology of executive functions: Catecholamine influences on prefrontal cortical functions. Biol. Psychiatry.

[43] Aghajanian GK, Sprouse JS, Sheldon P, Rasmussen K (1990). Electrophysiology of the central serotonin system: Receptor subtypes and transducer mechanisms. Ann. N. Y. Acad. Sci..

[44] Revelli A, Tesarik J, Massobrio M (1998). Nongenomic effects of neurosteroids. Gynecol. Endocrinol..

[45] Zhang Y, Wang W, Zhang J (2004). Effects of novel anxiolytic 4-butyl-alpha-agarofuran on levels of monoamine neurotransmitters in rats. Eur. J. Pharmacol..

[46] Thakare VN, Dhakane VD, Patel BM (2016). Potential antidepressant-like activity of silymarin in the acute restraint stress in mice: Modulation of corticosterone and oxidative stress response in cerebral cortex and hippocampus. Pharmacol. Rep..

[47] 47Wang, Y. L. The Study on Pharmacodynamics and Mechanism of Compound Ma Ti Xiang (CMTX). *Master's Thesis, Beijing University of Chinese Medicine, Beijing, China* (2011).

[48] Boutros N, Semenova S, Liu W, Crews FT, Markou A (2014). Adolescent intermittent ethanol exposure is associated with increased risky choice and decreased dopaminergic and cholinergic neuron markers in adult rats. Int. J. Neuropsychopharmacol..

[49] Boutros N (2018). Effects of adolescent alcohol exposure on stress-induced reward deficits, brain CRF, monoamines and glutamate in adult rats. Psychopharmacology.

[50] Wehry AM, Beesdo-Baum K, Hennelly MM, Connolly SD, Strawn JR (2015). Assessment and treatment of anxiety disorders in children and adolescents. Curr. Psychiatry Rep..

[51] Price JL, Drevets WC (2012). Neural circuits underlying the pathophysiology of mood disorders. Trends Cogn. Sci..

[52] Roche M, Kerr DM, Hunt SP, Kelly JP (2012). Neurokinin-1 receptor deletion modulates behavioural and neurochemical alterations in an animal model of depression. Behav. Brain Res..

[53] Motaghinejad M, Ghaleni MA, Motaghinejad O (2014). Preventive effects of forced exercise against alcohol-induced physical dependency and reduction of pain perception threshold. Int. J. Prev. Med..

[54] Cippitelli A (2010). Alcohol-induced neurodegeneration, suppression of transforming growth factor-beta, and cognitive impairment in rats: Prevention by group II metabotropic glutamate receptor activation. Biol. Psychiatry.

[55] Kempermann G, Wiskott L, Gage FH (2004). Functional significance of adult neurogenesis. Curr. Opin. Neurobiol..

[56] Imayoshi I (2008). Roles of continuous neurogenesis in the structural and functional integrity of the adult forebrain. Nat. Neurosci..

[57] Dworkin S, Mantamadiotis T (2010). Targeting CREB signalling in neurogenesis. Expert Opin. Ther. Targets.

[58] Ghitza UE (2010). Role of BDNF and GDNF in drug reward and relapse: A review. Neurosci. Biobehav. Rev..

[59] Briones TL, Woods J (2013). Chronic binge-like alcohol consumption in adolescence causes depression-like symptoms possibly mediated by the effects of BDNF on neurogenesis. Neuroscience.

[60] McClain JA, Morris SA, Marshall SA, Nixon K (2014). Ectopic hippocampal neurogenesis in adolescent male rats following alcohol dependence. Addict. Biol..

[61] Sakharkar AJ (2016). A role for histone acetylation mechanisms in adolescent alcohol exposure-induced deficits in hippocampal brain-derived neurotrophic factor expression and neurogenesis markers in adulthood. Brain Struct. Funct..

[62] Davis MI (2008). Ethanol-BDNF interactions: Still more questions than answers. Pharmacol. Ther..

[63] Passos IC (2015). Inflammatory markers in post-traumatic stress disorder: A systematic review, meta-analysis, and meta-regression. Lancet Psychiatry.

[64] Tucker P (2004). Neuroimmune and cortisol changes in selective serotonin reuptake inhibitor and placebo treatment of chronic posttraumatic stress disorder. Biol. Psychiatry.

[65] von Kanel R (2007). Evidence for low-grade systemic proinflammatory activity in patients with posttraumatic stress disorder. J. Psychiatr. Res..

[66] Goggi J, Pullar IA, Carney SL, Bradford HF (2003). The control of [125I]BDNF release from striatal rat brain slices. Brain Res..

[67] Zhang F, Lu YF, Wu Q, Liu J, Shi JS (2012). Resveratrol promotes neurotrophic factor release from astroglia. Exp. Biol. Med..

[68] Rasmussen P (2009). Evidence for a release of brain-derived neurotrophic factor from the brain during exercise. Exp. Physiol..

[69] Guo S (2008). Neuroprotection via matrix-trophic coupling between cerebral endothelial cells and neurons. Proc. Natl. Acad. Sci. USA..

[70] Ventriglia M (2013). Serum brain-derived neurotrophic factor levels in different neurological diseases. Biomed. Res. Int..

[71] Delerive P (1999). Peroxisome proliferator-activated receptor alpha negatively regulates the vascular inflammatory gene response by negative cross-talk with transcription factors NF-kappaB and AP-1. J. Biol. Chem..

[72] Finucane OM (2015). Monounsaturated fatty acid-enriched high-fat diets impede adipose NLRP3 inflammasome-mediated IL-1beta secretion and insulin resistance despite obesity. Diabetes.

[73] Faggiani E, Delaville C, Benazzouz A (2015). The combined depletion of monoamines alters the effectiveness of subthalamic deep brain stimulation. Neurobiol. Dis..

[74] Carobrez AP, Bertoglio LJ (2005). Ethological and temporal analyses of anxiety-like behavior: The elevated plus-maze model 20 years on. Neurosci. Biobehav. Rev..

[75] Konsman, J.-P. *The mouse brain in stereotaxic coordinates: Second Edition (Deluxe) By Paxinos G. and Franklin, K.B.J., Academic Press, New York, 2001, ISBN 0-12-547637-X*. Vol. 28 (2003).

[76] Moriya S, Tahara Y, Sasaki H, Ishigooka J, Shibata S (2015). Phase-delay in the light-dark cycle impairs clock gene expression and levels of serotonin, norepinephrine, and their metabolites in the mouse hippocampus and amygdala. Sleep Med..

[77] Koressaar T, Remm M (2007). Enhancements and modifications of primer design program Primer3. Bioinformatics.

[78] Untergasser A (2012). Primer3—new capabilities and interfaces. Nucleic Acids Res..

[79] Tahara Y (2015). Entrainment of the mouse circadian clock by sub-acute physical and psychological stress. Sci. Rep..

