# Supplementary material for: Gamma oryzanol impairs alcohol-induced anxiety-like behavior in mice via upregulation of central monoamines associated with Bdnf and Il-1β signaling
Source: Sci Rep. 2020 Jun 30;10:10677. doi: 10.1038/s41598-020-67689-w (PMC7326911; doi:10.1038/s41598-020-67689-w)
Supplement: Supplementary file 1 — Supplementary information [file 41598_2020_67689_MOESM1_ESM.pdf]

Gamma oryzanol impairs alcohol-induced anxiety-like behavior in mice via upregulation of central monoamines associated with *Bdnf* and *Il-1β* signaling

Author name and Affiliation

Salina Akter, Kazi Rasel Uddin, Hiroyuki Sasaki, Yijin Lyu, Shigenobu Shibata

Laboratory of Physiology and Pharmacology, School of Advanced Science and Engineering, Waseda University, Tokyo, Japan.

Supplemental Table S1 Summary results of statistical analysis

Figure	Test	Detail	Parametric or Non-parametric	Analysis	Factor	F value	P value
1 (A)	Body weight	W0 body weight (g)	Non-parametric	Kruskal-Wallis	-	-	0.1898
1 (A)	Body weight	W1 body weight (g)	Parametric	ANOVA			
				Factor1	Food	F (1, 41) = 3.845	0.1848
				Factor2	Drink	F (1, 41) = 0.3748	0.6759
				Interaction factor	Food × Drink	F (1, 41) = 0.1094	0.8212
1 (A)	Body weight	W2 body weight (g)	Parametric	ANOVA			
				Factor1	Food	F (1, 41) = 9.997	0.1486
				Factor2	Drink	F (1, 41) = 6.337	0.2479
				Interaction factor	Food × Drink	F (1, 41) = 0.1994	0.8363
1 (A)	Body weight	W3 body weight (g)	Parametric	ANOVA			
				Factor1	Food	F (1, 41) = 0.002959	0.9859
				Factor2	Drink	F (1, 41) = 0.02172	0.9619
				Interaction factor	Food × Drink	F (1, 41) = 0.626	0.7975
1 (A)	Body weight	W4 body weight (g)	Parametric	ANOVA			
				Factor1	Food	F (1, 41) = 0.06366	0.9472
				Factor2	Drink	F (1, 41) = 0.01126	0.9778
				Interaction factor	Food × Drink	F (1, 41) = 1.559	0.7432
1 (C)	Food intake	Food intake for 2 weeks (g)	Parametric	ANOVA			
				Factor1	Food	F (1, 13) = 0.003252	0.8597
				Factor2	Drink	F (1, 13) = 1.801	0.2025
				Interaction factor	Food × Drink	F (1, 13) = 0.09367	0.7644
1 (E)	Drink intake	Drink intake for 2 weeks (g)	Non-parametric	Kruskal-Wallis	-	-	0.5011
2 (A)	Elevated plus maze test	Distance travelled in the open arm (m)	Non-parametric	Kruskal-Wallis	-	-	0.0012
2 (B)	Elevated plus maze test	Time in the open arm (s)	Parametric	ANOVA			
				Factor1	Food	F (1, 41) = 4760	0.0106
				Factor2	Drink	F (1, 41) = 806.2	0.2770
				Interaction factor	Food × Drink	F (1, 41) = 10585	0.0003
2 (C)	Elevated plus maze test	No. of entries into the open arm	Parametric	ANOVA			
				Factor1	Food	F (1, 41) = 127.2	0.0567
				Factor2	Drink	F (1, 41) = 13.28	0.5300
				Interaction factor	Food × Drink	F (1, 41) = 117.7	0.0664
2 (D)	Elevated plus maze test	Distance travelled in the closed arm (m)	Parametric	ANOVA			
				Factor1	Food	F (1, 41) = 0.8924	0.3672
				Factor2	Drink	F (1, 41) = 0.001496	0.9704
				Interaction factor	Food × Drink	F (1, 41) = 0.7428	0.4103
2 (E)	Elevated plus maze test	Time in the closed arm (s)	Parametric	ANOVA			
				Factor1	Food	F (1, 41) = 6047	0.0450
				Factor2	Drink	F (1, 41) = 1079	0.3873
				Interaction factor	Food × Drink	F (1, 41) = 7886	0.0230
2 (F)	Elevated plus maze test	No. of entries into the closed arm	Non-parametric	Kruskal-Wallis	-	-	0.4439
3 (A)	HPLC	5-HT in hippocampus	Non-parametric	Kruskal-Wallis	-	-	0.0125
3 (B)	HPLC	5-HIAA in hippocampus	Non-parametric	Kruskal-Wallis	-	-	0.0002
3 (C)	HPLC	HVA in hippocampus	Non-parametric	Kruskal-Wallis	-	-	0.1429
3 (D)	HPLC	NE in hippocampus	Non-parametric	Kruskal-Wallis	-	-	0.5048
3 (E)	HPLC	MHPG in hippocampus	Non-parametric	Kruskal-Wallis	-	-	0.0027
3 (F)	HPLC	5-HT in cerebral cortex	Parametric	ANOVA			
				Factor1	Food	F (1, 49) = 0.5519	0.4611
				Factor2	Drink	F (1, 49) = 0.3085	0.5811
				Interaction factor	Food × Drink	F (1, 49) = 0.4373	0.5115
3 (G)	HPLC	5-HIAA in cerebral cortex	Non-parametric	Kruskal-Wallis	-	-	0.0087
3 (H)	HPLC	HVA in cerebral cortex	Non-parametric	Kruskal-Wallis	-	-	0.0061
3 (I)	HPLC	NE in cerebral cortex	Non-parametric	Kruskal-Wallis	-	-	0.5423
3 (J)	HPLC	MHPG in cerebral cortex	Non-parametric	Kruskal-Wallis	-	-	0.0223
4 (A)	RT-PCR	*Bdnf* expression in hippocampus	Non-parametric	Kruskal-Wallis	-	-	0.0118
4 (B)	RT-PCR	*Il1-β* expression in hippocampus	Parametric	ANOVA			
				Factor1	Food	F (1,37) = 6.438	0.0155
				Factor2	Drink	F (1,37) = 0.2536	0.6175
				Interaction factor	Food × Drink	F (1,37) = 4.723	0.0362
4 (C)	RT-PCR	*Bdnf* expression in cerebral cortex	Non-parametric	Kruskal-Wallis	-	-	0.0805
4 (D)	RT-PCR	*Il1-β* expression in cerebral cortex	Non-Parametric	Kruskal-Wallis	-	-	0.2073
5 (A)	ELISA	BDNF concentration in serum	Non-parametric	Kruskal-Wallis	-	-	0.4873
5 (B)	ELISA	IL1-β concentration in serum	Parametric	ANOVA			
				Factor1	Food	F (1,21) = 0.1322	0.6697
				Factor2	Drink	F (1,21) = 0.823	0.2926
				Interaction factor	Food × Drink	F (1,21) = 0.758	0.3119

Supplemental Table S2 Primer sequence for reverse transcription polymerase chain reaction

Gene		Sequences
Bdnf	Forward	5´-gcggcagataaaaaga-3´
	Reverse	5´-tcagttggcctttgga-3´
IL1-β	Forward	5′-tccatgagctttgtacaagg -3′
	Reverse	5′-ggtgctgatgtaccagttgg -3′
Gapdh	Forward	5´-tggtgaaggtcggtgtgaac-3´
	Reverse	5´-aatgaaggggtcgttgatgg-3´

Supplementary Fig. 1 Experiment Design

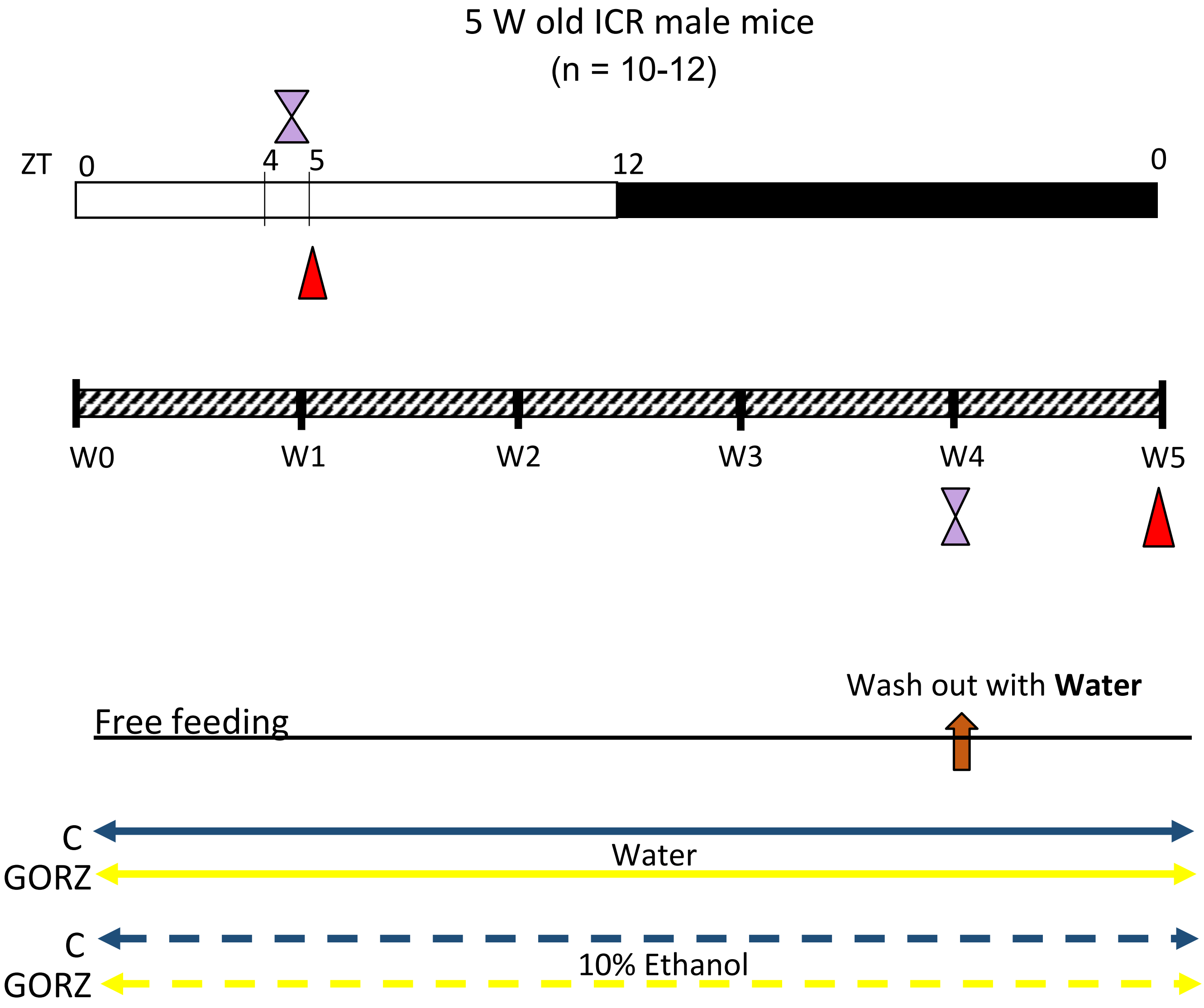

C (Control, 14% casein, AIN 93 M)
GORZ (γ-Oryzanol, 14% casein, AIN 93 M + 0.5% GORZ)
W (Week)
ZT (Zeitgeber time)

Sample: Blood, Hippocampus, cerebralcortex

Elevated plus maze test   Sampling
